# Changes in IgE sensitization and total IgE levels over 20 years of follow-up

**DOI:** 10.1016/j.jaci.2015.09.037

**Published:** 2016-06

**Authors:** André F.S. Amaral, Roger B. Newson, Michael J. Abramson, Josep M. Antó, Roberto Bono, Angelo G. Corsico, Roberto de Marco, Pascal Demoly, Bertil Forsberg, Thorarinn Gislason, Joachim Heinrich, Ismael Huerta, Christer Janson, Rain Jõgi, Jeong-Lim Kim, José Maldonado, Jesús Martinez-Moratalla Rovira, Catherine Neukirch, Dennis Nowak, Isabelle Pin, Nicole Probst-Hensch, Chantal Raherison-Semjen, Cecilie Svanes, Isabel Urrutia Landa, Ronald van Ree, Serge A. Versteeg, Joost Weyler, Jan-Paul Zock, Peter G.J. Burney, Deborah L. Jarvis

**Affiliations:** a Respiratory Epidemiology, Occupational Medicine and Public Health, National Heart and Lung Institute, Imperial College, London, United Kingdom; b Department of Primary Care and Public Health, School of Public Health, Imperial College, London, United Kingdom; c School of Public Health & Preventive Medicine, Monash University, Melbourne, Australia; d Centre for Research in Environmental Epidemiology (CREAL), Barcelona, Spain; e IMIM (Hospital del Mar Medical Research Institute), Barcelona, Spain; f Universitat Pompeu Fabra (UPF), Barcelona, Spain; g CIBER Epidemiología y Salud Pública (CIBERESP), Madrid, Spain; h Department of Public Health and Pediatrics, University of Turin, Turin, Italy; i Division of Respiratory Diseases, IRCCS Policlinico San Matteo Foundation–University of Pavia, Pavia, Italy; j Unit of Epidemiology and Medical Statistics, Department of Public Health and Community Medicine, University of Verona, Verona, Italy; k Department of Pulmonology, Division of Allergy, Arnaud de Villeneuve Hospital, CHU Montpellier, and EPAR Team–UMR-S 1136 INSERM, Paris, France; l Division of Occupational and Environmental Medicine, Department of Public Health and Clinical Medicine, Umeå University, Umeå, Sweden; m Faculty of Medicine, University of Iceland, Reykjavik, Iceland; n Department of Respiratory Medicine and Sleep, Landspitali–The National University Hospital of Iceland, Reykjavik, Iceland; o Institute of Epidemiology I, Helmholtz Zentrum, Munich, Germany; p Institute and Outpatient Clinic for Occupational, Social and Environmental Medicine, Inner City Clinic, University Hospital Munich, Ludwig-Maximilians-Universität of Munich, Munich, Germany; q Epidemiological Surveillance Section, Directorate General of Public Health, Department of Health of Asturias, Oviedo, Spain; r Department of Medical Sciences: Respiratory, Allergy and Sleep Research, Uppsala University, Uppsala, Sweden; s Tartu University Hospital, Lung Clinic, Tartu, Estonia; t Department of Publich Health and Community Medicine, Sahlgrenska Academy, University of Gothenburg, Gothenburg, Sweden; u Unit of Clinical Management of Pneumology and Allergy, University Hospital of Huelva, Huelva, Spain; v Unit of Pneumology, University Hospital of Albacete, Albacete, Spain; w INSERM UMR1152, Paris, France; x Université Paris Diderot Paris 7, UMR1152, Paris, France; y Institute and Outpatient Clinic for Occupational, Social and Environmental Medicine, Inner City Clinic, University Hospital Munich, Ludwig-Maximilians-Universität of Munich, and the German Center for Lung Research, Munich, Germany; z Pédiatrie, Pole Couple Enfants, CHU de Grenoble, Grenoble, France; aa INSERM U823, Institut Albert Bonniot, Grenoble, France; bb Université Joseph Fourier, Grenoble, France; cc Swiss Tropical and Public Health Institute, Basel, Switzerland; dd University of Basel, Basel, Switzerland; ee INSERM U897, Institute of Public health and Epidemiology, Bordeaux University, Bordeaux, France; ff Centre for International Health, University of Bergen, Bergen, Norway; gg Department of Occupational Medicine, Haukeland University Hospital, Bergen, Norway; hh Department of Pneumology, Galdakao Hospital, Galdakao, Spain; ii Departments of Experimental Immunology and of Otorhinolaryngology, Academic Medical Centre, University of Amsterdam, Amsterdam, The Netherlands; jj Department of Experimental Immunology, Academic Medical Centre, University of Amsterdam, Amsterdam, The Netherlands; kk Epidemiology and Social Medicine and the StatUA Statistics Centre, University of Antwerp, Antwerp, Belgium

**Keywords:** Allergens, sensitization, cohort study, epidemiology, IgE, longitudinal analysis, aging, immunosenescence, ECRHS, European Community Respiratory Health Survey, GM, Geometric mean

## Abstract

**Background:**

Cross-sectional studies have reported a lower prevalence of sensitization in older adults, but few longitudinal studies have examined whether this is an aging or a year-of-birth cohort effect.

**Objective:**

We sought to assess changes in sensitization and total IgE levels in a cohort of European adults as they aged over a 20-year period.

**Methods:**

Levels of serum specific IgE to common aeroallergens (house dust mite, cat, and grass) and total IgE levels were measured in 3206 adults from 25 centers in the European Community Respiratory Health Survey on 3 occasions over 20 years. Changes in sensitization and total IgE levels were analyzed by using regression analysis corrected for potential differences in laboratory equipment and by using inverse sampling probability weights to account for nonresponse.

**Results:**

Over the 20-year follow-up, the prevalence of sensitization to at least 1 of the 3 allergens decreased from 29.4% to 24.8% (−4.6%; 95% CI, −7.0% to −2.1%). The prevalence of sensitization to house dust mite (−4.3%; 95% CI, −6.0% to −2.6%) and cat (−2.1%; 95% CI, −3.6% to −0.7%) decreased more than sensitization to grass (−0.6%; 95% CI, −2.5% to 1.3%). Age-specific prevalence of sensitization to house dust mite and cat did not differ between year-of-birth cohorts, but sensitization to grass was most prevalent in the most recent ones. Overall, total IgE levels decreased significantly (geometric mean ratio, 0.63; 95% CI, 0.58-0.68) at all ages in all year-of-birth cohorts.

**Conclusion:**

Aging was associated with lower levels of sensitization, especially to house dust mite and cat, after the age of 20 years.

Population-based cross-sectional studies have shown that the prevalence of sensitization is higher in younger than in older age groups.^1^, ^2^, ^3^, ^4^ Although there have been year-of-birth cohort-related increases in atopy over the last decades, it is hypothesized that these cross-sectional observations might reflect decreases in sensitization with aging-related immunosenescence. Longitudinal studies that have performed skin prick tests or measured serum allergen-specific IgE levels at baseline and follow-up over periods of up to 14 years have reported that sensitization increased with aging, although changes were less evident in middle-aged and older adults.^2^, ^5^, ^6^, ^7^ Two recent longitudinal studies reported no change or a slight decrease in sensitization with aging.^4^, ^8^ In one of these studies, changes in sensitization were based on allergen-specific IgE measures,^8^ whereas in the other the comparison between time points was based on both specific IgE levels and skin prick test responses.^4^

Within the European Community Respiratory Health Survey (ECRHS),^9^ a multicenter cohort study of more than 6000 young and middle-aged adults followed for a 10-year period, there was little evidence of substantial change in sensitization to at least 1 of cat, grass, or house dust mite (as measured based on serum specific IgE levels) over time as the cohort aged. The age-specific prevalence of sensitization to grass but not to the other allergens measured was higher in more recent year-of-birth cohorts. At the time, it was observed that changes in laboratory methods between baseline and follow-up could influence assessment of change in sensitization; such biases are even more difficult to quantify when using skin prick tests.

Completion of the third phase of the ECRHS has allowed assessment of serum specific IgE levels on 3 occasions: baseline and 10- and 20-year follow-up. The aims of this report were to (1) assess the changes in IgE sensitization and total IgE levels in this population-based cohort of European adults over a period of 20 years and (2) to investigate whether these changes were different between year-of-birth cohorts.

## Methods

### Study participants

This is a multicenter population-based cohort study. Detailed descriptions of the methods for ECRHS I and ECRHS II have been published elsewhere.^10^, ^11^ In ECRHS I 1500 men and 1500 women aged 20 to 44 years were randomly recruited from community-based sampling frames in each center. After completing a short postal screening questionnaire, a random sample of responders was selected to complete an interviewer-led questionnaire and provided a blood sample (1991-1993). In the majority of centers, an additional sample of patients with symptoms highly suggestive of asthma were recruited for the study, but these participants are not included in the present analysis.

In ECRHS II (1998-2002) participants who had completed the extended questionnaire in ECRHS I were reinvestigated and again provided a blood sample. In ECRHS III those who took part in the clinical stages of ECRHS I and II were again contacted, with responders invited to a local testing center where blood samples were taken once more (2008-2013).

Eleven countries are represented in this report: Iceland (Reykjavik), Norway (Bergen), Sweden (Gothenburg, Umeå, and Uppsala), Estonia (Tartu), Belgium (Antwerp South and Antwerp City), Germany (Hamburg and Erfurt), the United Kingdom (Ipswich and Norwich), France (Bordeaux, Grenoble, Montpelier, and Paris), Spain (Barcelona, Galdakao, Albacete, Oviedo, and Huelva), Italy (Pavia, Turin, and Verona), and Australia (Melbourne).

Ethical approval for the study from local research ethics committees and written consent from participants were obtained.

### Measurement of IgE levels

In all 3 surveys blood samples were obtained and processed under similar conditions. After clotting and centrifuging, serum was stored at −20°C until analysis in a single central laboratory (Pharmacia Uppsala in 1992, Kings College London in 2002, and AMC Amsterdam in 2013/2014) by using the Phadia ImmunoCAP system (now Thermo Fisher Scientific, Uppsala, Sweden).

To assess the effects of potential laboratory bias on the prevalence of IgE sensitization and the mean of total IgE estimates, we conducted duplicate assays on 794 samples (tested at ECRHS I, stored, and tested at ECRHS II) and 475 samples (tested at ECRHS II, stored, and tested at ECRHS III; see Table E1 in this article's Online Repository at www.jacionline.org). The methods for this correction are described in detail in the Methods section in this article's Online Repository at www.jacionline.org.

### Outcomes

Participants were considered sensitized if allergen-specific IgE to *Dermatophagoides pteronyssinus* (house dust mite), *Felis silvestris catus* (cat), and *Phleum pratense* (Timothy grass) was present in concentrations of greater than 0.35 kU_A_/L. A higher threshold (>0.70 kU_A_/L) was also considered. *Atopy* was defined as being sensitized to 1 of either house dust mite, grass, or cat. Total IgE, expressed in kilounits/liters, was log-transformed and considered as a continuous outcome for estimation of geometric means (GMs) and their ratios.

### Statistical methods

**Fig 1:**
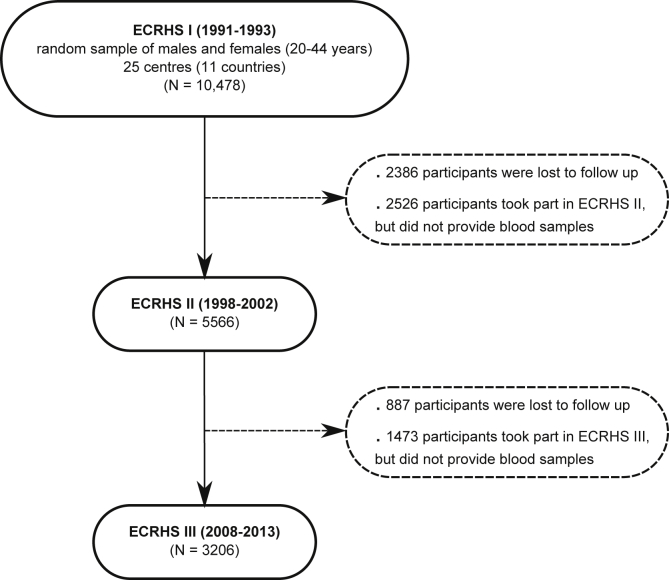
Participant flow in the ECRHS. Only centers that took part in all 3 surveys are included.

Statistical analyses were performed with Stata software (version 13; StataCorp LP, College Station, Tex). Analyses were restricted to the 3206 participants with information on serum specific IgE and total IgE levels in all 3 ECRHSs (Fig 1). Inverse sampling probability weights were used to standardize the estimation from this population with data on IgE assays from all 3 ECRHSs to the original target population of participants with data on IgE assays from ECRHS I (see the Methods section in this article's Online Repository for details on the inverse sampling probability weighted estimation).

The prevalence of sensitization at each survey was determined by using logistic regression with Huber variances considering participants as the clusters. CIs for prevalences and their differences (net change) between ECRHS II and I, ECRHS III and II, and ECRHS III and I were estimated by using the normalizing hyperbolic arctangent transformation.^12^ Similarly, by using linear regression, we calculated GM ratios of total IgE levels between ECRHS II and I, ECRHS III and II, and ECRHS III and I.

Statistical analyses for each outcome were performed in 2 ways by using uncorrected models and models corrected for potential laboratory bias. Only results of the corrected models are presented in this report. Because data came from multiple centers, we tested for between-center heterogeneity in the uncorrected results by using the methods of Cochran.^13^

In a final step analyses were repeated as follows: (1) stratified by sex; (2) restricted to lifetime nonsmokers; and (c) stratified by year-of-birth cohort. For this latter step, year-of-birth cohorts were defined by date of birth (1964-1973, 1954-1963, and 1944-1953). The ages of these participants at January 1, 1992 (the approximate midpoint of ECRHS I data collection), would have been as follows: 18 years ≤ age < 28 years, 28 years ≤ age < 38 years, and 38 years ≤ age ≤ 48 years, respectively. Participants from Tartu, Estonia, were recruited at age 20-44 years in 1994 and would have been less than 20 years old on January 1, 1992; hence 18 years is the lower age limit. Members of each age cohort would have been 10 years older on January 1, 2002 (during the ECRHS II data collection), and 20 years older on January 1, 2012 (during the ECRHS III data collection). This approach allowed comparison of earlier cohorts with later cohorts at approximately the same ages.

## Results

A total of 3,206 (30.6%) of the 10,478 participants who provided a blood sample in the first survey took part and again provided a sample in both ECRHS II and III. The median age of participants in ECRHS I was 34.9 years (interquartile range, 28.6-40.5 years), half were males, and forty-five percent were lifetime nonsmokers. There was variation between centers in the proportion of participants who provided samples at ECRHS I and then went on to provide samples at ECRHS II and ECRHS III (minimum, 13.6% in Pavia; maximum, 58.6% in Reykjavik). Factors associated with response were older age and being a nonsmoker. Response was not associated with sensitization at baseline, sex, and reporting of wheeze (see Table E2 in this article's Online Repository at www.jacionline.org), although those who took part in all 3 surveys reported waking with breathlessness less frequently.

### Net change in IgE sensitization and total IgE levels

Laboratory-corrected net changes in the prevalence of IgE sensitization to each of the allergens and in GMs of total IgE levels over a period of 20 years are shown in Table I. Between ECRHS I and ECRHS II, there was no significant change in the prevalence of IgE sensitization to any of the allergens by using either the low or high cutoff levels.

Over the 20 years of follow-up (ie, between ECRHS I and ECRHS III), the prevalence of IgE sensitization to house dust mite, cat, and at least 1 allergen decreased. By using the 0.35 kU_A_/L cutoff, the prevalence of sensitization to grass remained stable, but when the 0.70 kU_A_/L cutoff was used, there was evidence of a reduction in sensitization. These changes were similar in men and women (see Table E3 in this article's Online Repository at www.jacionline.org).

**Fig E1:**
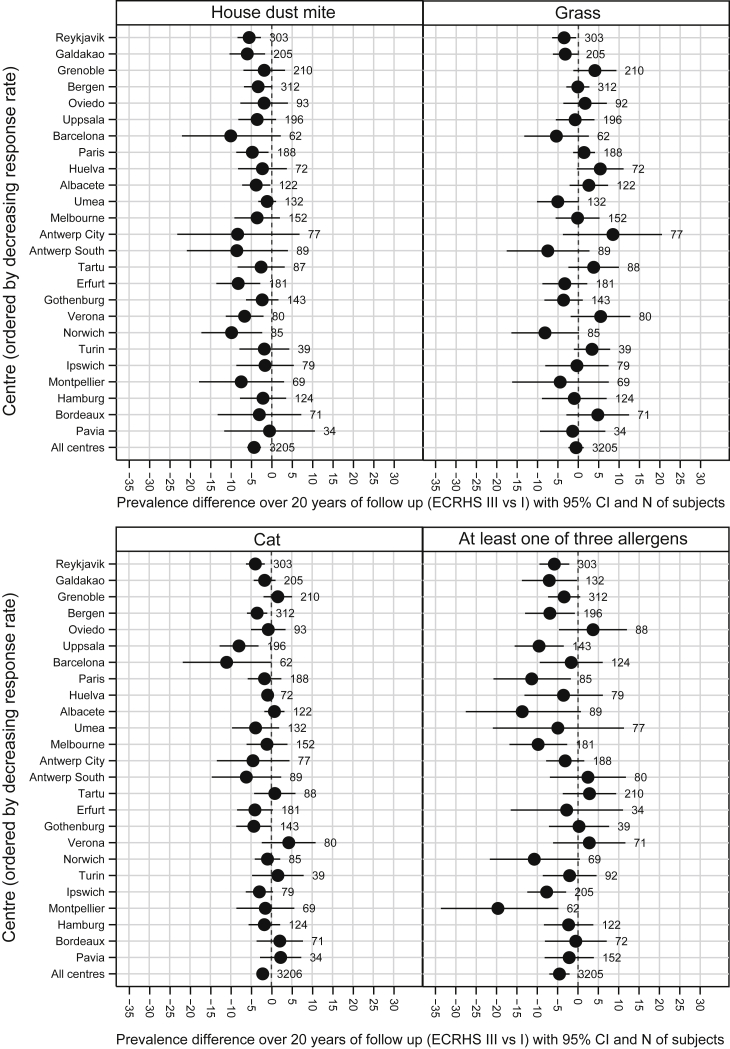
Net change in prevalence of IgE sensitization (cutoff, 0.35 kU_A_/L) to house dust mite, grass, cat, and at least 1 of these allergens. Centers are sorted by descending response rate.

**Fig E2:**
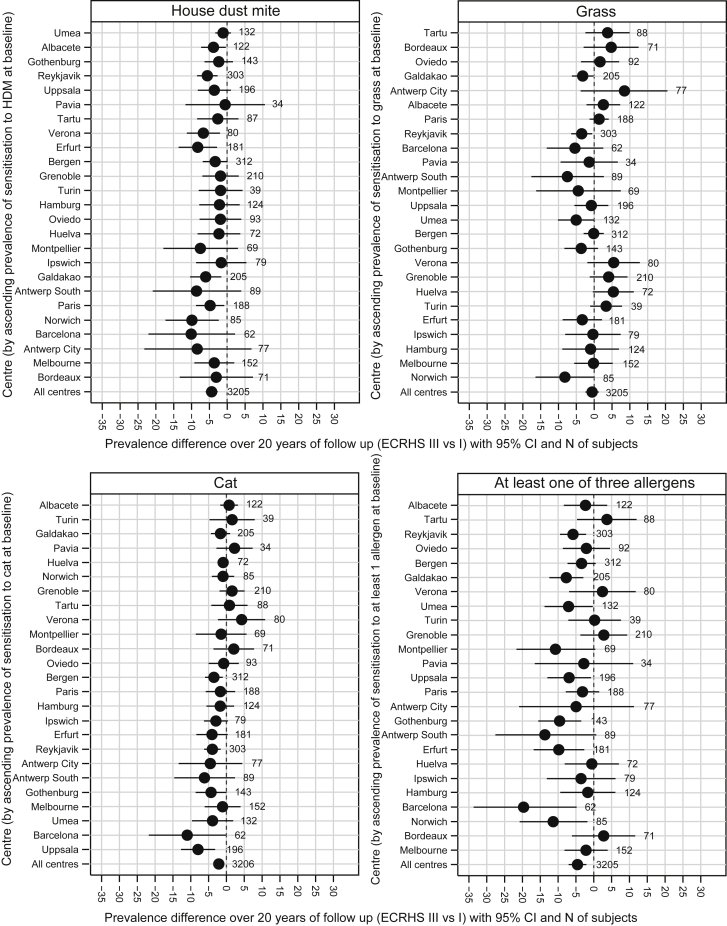
Net change in prevalence of IgE sensitization (cutoff, 0.35 kU_A_/L) to house dust mite, grass, cat, and at least 1 of these allergens. Centers are sorted by ascending prevalence of sensitization at baseline.

**Fig 2:**
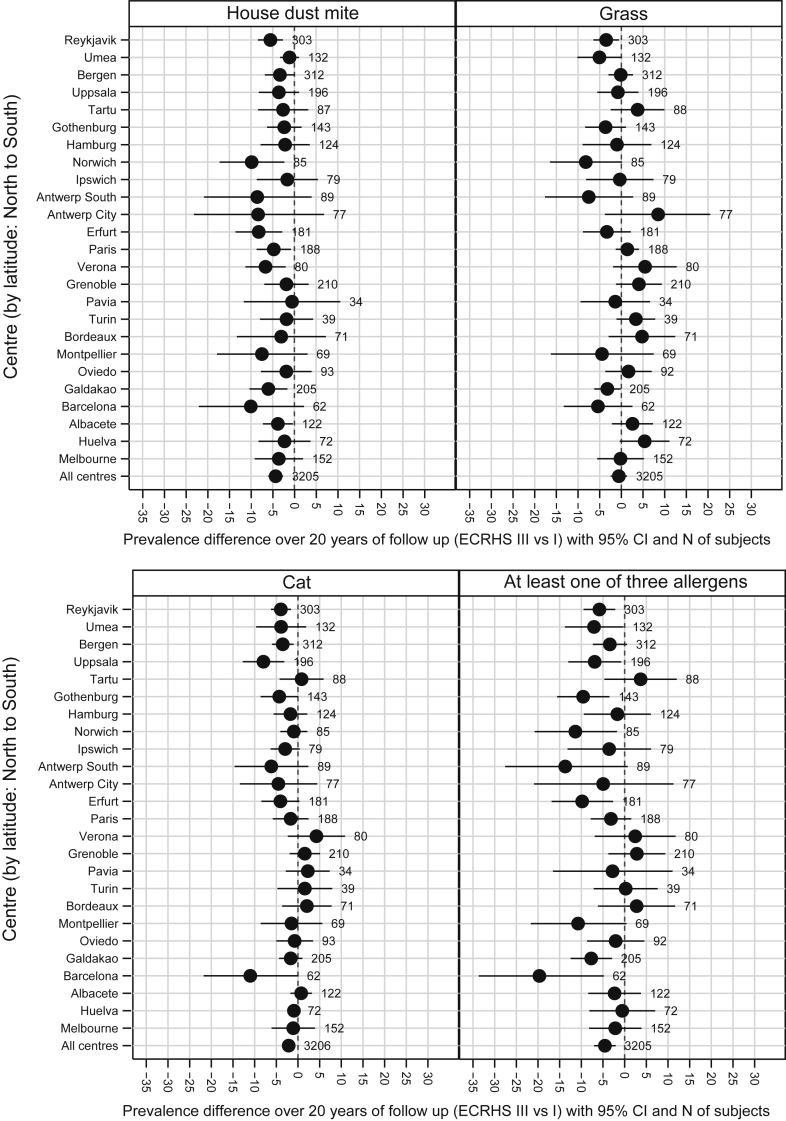
Net change in prevalence of IgE sensitization (cutoff, 0.35 kU_A_/L) to house dust mite (*I*^*2*^ [heterogeneity] = 0.0%, *P* = .71), grass (*I*^*2*^ = 44.9%, *P* = .009), cat (*I*^*2*^ = 29.0%, *P* = .09), and at least 1 of these allergens (*I*^*2*^ = 38.6%, *P* = .03). Centers are sorted by latitude (from north to south).

For some estimates, there was evidence of heterogeneity between countries, but no clear pattern in this variation was observed by latitude (Fig 2), response rate (see Fig E1 in this article's Online Repository at www.jacionline.org), or prevalence of sensitization at baseline (see Fig E2 in this article's Online Repository at www.jacionline.org).

**Fig E3:**
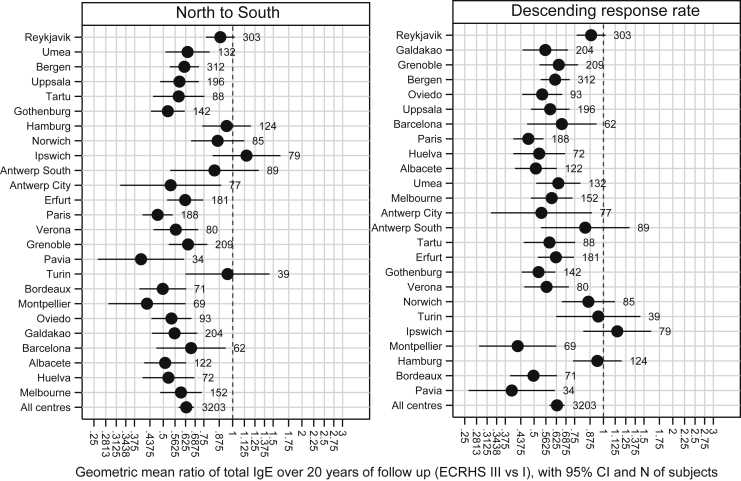
Net change in GM ratio of total IgE levels (kilounits per liter). Centers were sorted by latitude (north to south; *left*) and descending response rate *(right)*.

Overall, there was a significant decrease in total IgE levels over the 20 years of follow-up (GM ratio, 0.63; 95% CI, 0.58-0.68). This generalized decrease in total IgE levels occurred in all centers, although the magnitude of the change varied (heterogeneity between centers, *P* < .001; see Fig E3 in this article's Online Repository at www.jacionline.org). Patterns were similar in men and women (see Table E3).

Restriction of analyses to the 1304 participants who were lifetime nonsmokers did not materially alter the results reported above (see Table E4 in this article's Online Repository at www.jacionline.org).

### Association of net change with age and cohort

In ECRHS I the prevalence of IgE sensitization to house dust mite, grass, cat, and at least 1 allergen was higher in younger adults (ie, those born more recently) than in older adults (Table II).

**Fig 3:**
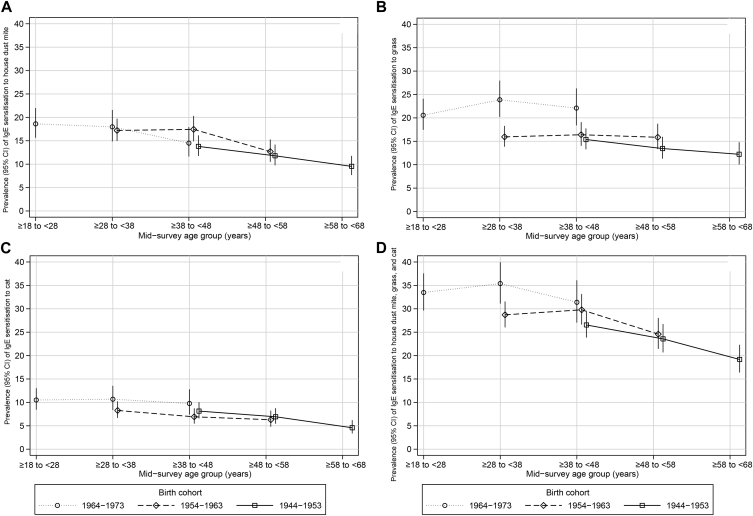
Prevalence of IgE sensitization to house dust mite **(A)**, grass **(B)**, cat **(C)**, and at least 1 of these 3 allergens **(D)** over 20 years of follow-up by year-of-birth cohort.

Over the 20-year period, the prevalence of sensitization to house dust mite decreased in all age groups to a similar extent, and there was little evidence that the age-specific prevalence of sensitization to house dust mite was different between those born more recently and those born earlier (Fig 3, *A*). Overall, the picture was one of a decrease in sensitization with age, with decreases occurring throughout adult life. This was broadly similar for sensitization to cat (Fig 3, *C*). However, these patterns were different for sensitization to grass. Although there was evidence of a decrease in sensitization to grass in those who were the oldest at recruitment (ie, the earlier cohort), decreases were not seen in those who were born more recently. As a result, there were marked differences in the age-specific prevalence of sensitization to grass between cohorts with higher age-specific prevalence in those born after 1964 (Fig 3, *B*). The prevalence of IgE sensitization to at least 1 of house dust mite, grass, and cat showed a pattern similar to that of sensitization to house dust mite and cat. The most recent cohort had the highest prevalence at younger ages, but these cohort-related differences were not apparent in later adult life (Fig 3, *D*). Similar patterns were observed when using the cutoff of 0.70 kU_A_/L (see Table E5 in this article's Online Repository at www.jacionline.org).

**Fig 4:**
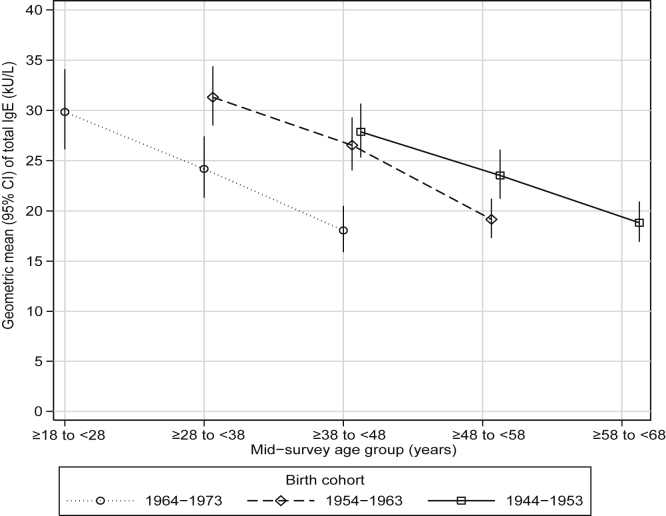
Changes in total IgE levels (kilounits per liter) over 20 years of follow-up by year-of-birth cohort.

The population GM of total IgE was lower at each follow-up in all cohorts over the 20-year period of follow-up, and the more recent cohorts had lower total IgE levels than those born earlier at the equivalent ages (Fig 4 and Table II).

## Discussion

We have shown that the prevalence of sensitization to at least 1 of house dust mite, cat, or grass has decreased within a large population-based adult cohort followed over a period of 20 years. There was a decrease in the prevalence of sensitization to house dust mite and cat, and the GM total IgE levels also decreased. Sensitization to grass did not follow these patterns so clearly, showing instead an increase at younger ages and aging effects only at older ages.

The strengths of this study are the population-based nature of the sample derived from several parts of Europe and Australia, the prolonged period of follow-up, and the standardized handling and testing of samples between centers and over time. Changes in laboratory staff, consumables, and methods between surveys could lead to bias in prevalence estimates, and to address this, we have used information from duplicate assays of hundreds of samples to adjust our estimates. As with all cohorts, there has been attrition during the 20-year period of follow-up, and the analyses we present are based on participants who have taken part in all 3 phases of the study. We are aware that considerable loss to follow-up has the potential to induce bias, and therefore to account for small differences between these subjects and the initial cohort at baseline and to enhance the external validity of our results, we have corrected our models with inverse sampling probability weights. This method generates estimates that apply to the population we sampled at baseline. We are unable to say whether the start of the age-related decrease in sensitization occurs around the age of 20 years or earlier because the ECRHS is a cohort of adults only.

To date, few other population-based studies have reported on longitudinal changes in sensitization by measuring serum specific IgE levels.^6^, ^8^ These earlier reports, both from Denmark, are of smaller samples and mostly over shorter time periods. Linneberg et al^6^ studied changes over an 8-year period in serum specific IgE levels to at least 1 of 6 allergens in about 400 adolescents and adults in Copenhagen, reporting an increase in the prevalence of IgE sensitization, especially among those born in the 1960s or later. Older adults (>40 years, n = 695) living in the same city and followed for 20 years showed no change in sensitization over a 20-year period in prevalence of IgE sensitization to at least 1 of 19 allergens.^8^ Other studies looked at changes in sensitization by performing skin prick tests and reported increases with aging.^2^, ^4^, ^5^ However, skin prick tests are much more difficult to standardize over different periods because they are prone to fieldworker variation, with changes in skin prick test reagents being difficult to assess.^14^, ^15^

Barbee et al^16^ studied 1100 participants in the United States and reported a decrease in total IgE levels with age in children and young adults but not in older adults. In ECRHS total IgE levels decreased with aging within each cohort, with more recent cohorts having lower total IgE levels than earlier ones at the same age. In a previous report we showed that smoking associated differently with sensitization to different aeroallergens and in a dose-response manner with total IgE levels.^17^ Therefore we hypothesized that changes in sensitization over time could be related to decreasing smoking rates and that lifetime nonsmokers would not show changes in sensitization. Our present findings show that a decrease in sensitization is unlikely to be related to smoking cessation. The decrease in total IgE levels in our study might in part be explained by a decrease in helminth infestation, as observed by others in children.^18^

We saw no evidence of change in the prevalence of IgE sensitization to house dust mite, cat, grass, and at least 1 of these 3 as the cohort aged over the initial 10 years of follow-up of the ECRHS.^9^ This observation is confirmed within this second report, but we go on to show that prevalence does decrease over 20 years and appears greater when subjects are aged about 40 years or older. This finding might be explained by immunosenescence, which seems to be more evident after 50 years of age^19^ and corresponds to age-related changes in the number and function of cells from the immune system.^20^ The production of IgE, which is dependent on an interaction between B and T cells,^21^ might decrease as a consequence of the naturally occurring involution of the thymus^22^; the thymic output of T cells per day in a 50-year-old is about 33% lower than that in a 25-year-old.^22^ Our findings are supported by animal studies, which suggest that the production of IgE to an allergen challenge is higher in younger than older animals.^23^, ^24^ In one of these studies, the transplantation of thymocytes into young (8 weeks old) mice resulted in no change in IgE response, whereas that into aged (65 weeks old) mice resulted in an enhanced IgE response similar to that into young mice.^24^

One might expect all markers of atopy to follow similar age/period/cohort patterns. Our report suggests house dust mite and cat might be different to grass, but we can only speculate as to the reason for this. One explanation for the decrease in sensitization to house dust mite and cat could be avoidance by the participants. We cannot assess whether participants avoided house dust mite allergen, but we do know that the prevalence of cat ownership among those with IgE at all 3 time points has not decreased over the 20 years of follow-up (16.9% at ECRHS I and 19.5% at ECRHS III). This supports the hypothesis that the decrease in prevalence of sensitization to cat is more likely due to aging-related immunosenescence. There are differences in the epidemiology of sensitization to each of the 3 allergens, particularly with respect to factors associated with the hygiene hypothesis. Larger sibships protect younger siblings from hay fever and sensitization to grass more strongly than from asthma and sensitization to house dust mites.^25^, ^26^ Decreasing family size over the last decades might explain the less marked aging effect for grass than for other allergens. Changes in the level of exposure to pollens might have had a role in our findings.^27^, ^28^ There are also reports suggesting that pollens in our more modern society are more allergenic than they have been previously,^29^, ^30^ which could be related to the high levels of air pollutants, such as ozone, nitrogen dioxide, and carbon dioxide.^30^, ^31^, ^32^ The presence of unmeasured factors might also have a role in the different patterns observed in sensitization to the 3 allergens.

In summary, over a period of 20 years, the prevalence of specific IgE sensitization to house dust mite and cat, but not grass, significantly decreased in the multinational cohort of adults from the ECRHS as a consequence of aging, being more evident among those aged 40 years or older.

**Key messages**

• Allergen-specific and total IgE levels decrease after the age of 20 years as subjects become older.
• Kinetics of IgE sensitization decrease differently for different allergens and might be faster after 40 years of age.
• The biological mechanism and environmental determinants for IgE sensitization that decrease with aging need to be explored so that we can improve our understanding of the cause of atopy and atopic diseases.

## Figures and Tables

**Table E1:** Results from a comparability study in which replicate samples from 1992 were tested in 2002 and replicate samples from 2002 were tested in 2013/2014

	IgE in 1992		IgE in 2002		Difference (%), 2002 vs 1992 (95% CI), n = 794	Cohen κ, 2002 vs 1992	IgE in 2002		IgE in 2013/2014		Difference (%), 2013/2014 vs 2002 (95% CI), n = 475	Cohen κ, 2013/2014 vs 2002
No. (of 794)	Percent	No. (of 794)	Percent	No. (of 475)	Percent	No. (of 475)	Percent
House dust mite												
0.35 kU_A_/L	241	30.4	247	31.1	0.8 (−1.3 to 2.8)	0.80	129	27.2	133	28.0	0.8 (−0.6 to 2.3)	0.94
0.70 kU_A_/L	193	24.3	195	24.6	0.3 (−1.1 to 1.6)	0.89	106	22.3	104	21.9	−0.4 (−1.4 to 0.6)	0.96
Grass												
0.35 kU_A_/L	229	28.8	224	28.2	−0.6 (−2.3 to 1.1)	0.86	119	25.1	115	24.2	−0.8 (−2.1 to 0.5)	0.94
0.70 kU_A_/L	187	23.6	196	24.7	1.1 (−0.3 to 2.6)	0.88	99	20.8	98	20.6	−0.2 (−1.6 to 1.2)	0.93
Cat												
0.35 kU_A_/L	116	14.6	133	16.8	2.1 (0.7 to 3.6)	0.83	60	12.6	63	13.3	0.6 (−0.7 to 2.0)	0.90
0.70 kU_A_/L	94	11.8	102	12.8	1.0 (−0.3 to 2.3)	0.85	51	10.7	54	11.4	0.6 (−0.5 to 1.7)	0.92
Sensitization to ≥1 allergen												
0.35 kU_A_/L	336	42.3	338	42.6	0.3 (−1.8 to 2.3)	0.82	182	38.3	186	39.2	0.8 (−0.9 to 2.6)	0.92
0.70 kU_A_/L	278	35.0	293	36.9	1.9 (0.4 to 3.4)	0.89	159	33.5	162	34.1	0.6 (−0.7 to 2.0)	0.95

	GM in 1992, n = 794	GM in 2002, n = 794	GM ratio, 2002 vs 1992 (95% CI)	GM in 2002, n = 475	GM in 2013/2014, n = 475	GM ratio, 2013/14 vs 2002 (95% CI)
Total IgE (kU/L)	36.1	52.75	1.46 (1.38-1.55)	42.7	43.2	1.01 (0.98-1.05)

**Table E2:** Baseline characteristics of subjects with IgE measurements in all 3 ECRHSs versus subjects with IgE measurements in baseline survey only from same centers

	With IgE measurements in baseline survey only (n = 7272)	With IgE measurements in all 3 surveys (n = 3206)	Adjusted∗ odds for responding (95% CI)	*P* value for heterogeneity‡
Age at baseline (per 10 y)	—	—	1.40 (1.29-1.52)	.036
Female sex (%)	49.9	50.0	1.00 (0.19-1.11)	.17
Smoking status at baseline (%)				
Lifetime nonsmoker	41.6	45.1	1.00	
Exsmoker	21.1	22.6	0.88 (0.78-1.01)	.29
Current smoker	37.3	32.3	0.65 (0.58-0.73)	.38
Symptoms in the last 12 mo				
Wheeze	22.2	19.8	0.97 (0.84-1.11)	.12
Woken with shortness of breath	6.4	4.8	0.76 (0.61-0.94)	.40
Sensitized to ≥1 allergen† (%)	29.5	27.9	1.05 (0.91-1.22)	.0017

∗ From meta-analysis by center, adjusting for all other factors in the table.

† House dust mite, cat, or grass.

‡ From random-effects meta-analysis.

**Table E3:** Net change in IgE sensitization to house dust mite, grass, and cat and total IgE levels over 20 years by sex

	Male subjects (n = 1604)					Female subjects (n = 1602)				
Prevalence (%), ECRHS I	Net change (95% CI), ECRHS II vs I	*P* value for heterogeneity between centers	Net change (95% CI), ECRHS III vs I	*P* value for heterogeneity between centers	Prevalence (%), ECRHS I	Net change (95% CI), ECRHS II vs I	*P* value for heterogeneity between centers	Net change (95% CI), ECRHS III vs I	*P* value for heterogeneity between centers
House dust mite										
>0.35 kU_A_/L	19.7	−0.5 (−2.7 to 1.6)	.20	−5.0 (−7.2 to −2.8)	.59	13.5	−0.8 (−2.5 to 0.9)	.038	−3.7 (−5.7 to −1.7)	.34
>0.70 kU_A_/L	15.1	−0.3 (−2.0 to 1.4)	.95	−2.9 (−4.9 to −0.9)	.26	11.0	−1.1 (−2.3 to 0.1)	.096	−3.3 (−5.0 to −1.6)	.057
Grass										
>0.35 kU_A_/L	18.5	0.4 (−1.6 to 2.4)	.18	−0.9 (−3.2 to 1.3)	.11	15.6	0.6 (−1.2 to 2.4)	.94	−0.2 (−2.5 to 2.1)	.74
>0.70 kU_A_/L	15.8	−0.3 (−2.0 to 1.5)	.16	−3.1 (−5.1 to −1.0)	.82	12.7	0.3 (−1.2 to 1.8)	.91	−1.3 (−3.3 to 0.6)	.95
Cat										
>0.35 kU_A_/L	8.7	−0.3 (−1.9 to 1.3)	.21	−2.1 (−3.8 to −0.4)	.40	8.9	−1.5 (−2.9 to −0.1)	.54	−2.2 (−3.9 to −0.5)	.074
>0.70 kU_A_/L	6.4	0.2 (−1.2 to 1.6)	.22	−1.2 (−2.7 to 0.3)	.27	6.4	−0.1 (−1.4 to 1.1)	.071	−1.0 (−2.3 to 0.4)	.013
House dust mite, grass, or cat										
>0.35 kU_A_/L	32.5	0.8 (−1.8 to 3.5)	.74	−5.6 (−8.6 to −2.5)	.39	26.2	−0.7 (−3.0 to 1.6)	.46	−3.6 (−6.4 to −0.7)	.089
>0.70 kU_A_/L	26.5	0.3 (−2.0 to 2.5)	.81	−4.6 (−7.2 to −2.0)	.25	21.9	−1.5 (−3.2 to 0.3)	.40	−4.5 (−6.8 to −2.2)	.056

	GM, ECRHS I	GM ratio (95% CI), ECRHS II vs I	*P* value for heterogeneity between centers	GM ratio (95% CI), ECRHS III vs I	*P* value for heterogeneity between centers	GM, ECRHS I	GM ratio (95% CI), ECRHS II vs I	*P* value for heterogeneity between centers	GM ratio (95% CI), ECRHS III vs I	*P* value for heterogeneity between centers
Total IgE (kU/L)	34.3	0.82 (0.75 to 0.88)	<.001	0.65 (0.59 to 0.71)	<.001	26.0	0.86 (0.79 to 0.93)	.004	0.61 (0.56 to 0.67)	<.001

**Table E4:** Net change in IgE sensitization to house dust mite, grass, and cat and total IgE levels over 20 years: persistent lifetime nonsmokers only (n = 1304)

	Prevalence (%), ECRHS I	Net change (95% CI), ECRHS II vs I	*P* value for heterogeneity between centers	Net change (95% CI), ECRHS III vs I	*P* value for heterogeneity between centers
House dust mite					
>0.35 kU_A_/L	15.8	0.0 (−1.9 to 2.0)	.005	−3.4 (−5.5 to −1.4)	.08
>0.70 kU_A_/L	12.4	−0.9 (−2.2 to 0.5)	.79	−2.0 (−3.8 to −0.2)	.41
Grass					
>0.35 kU_A_/L	20.5	1.1 (−1.0 to 3.3)	.75	−0.4 (−3.0 to 2.2)	.26
>0.70 kU_A_/L	17.9	0.2 (−1.6 to 2.1)	.65	−2.5 (−4.9 to −0.1)	.98
Cat					
>0.35 kU_A_/L	10.5	−0.6 (−2.3 to 1.1)	.78	−2.0 (−4.1 to 0.0)	.42
>0.70 kU_A_/L	8.0	0.4 (−1.2 to 2.0)	.71	−0.8 (−2.5 to 1.0)	.42
House dust mite, grass, or cat					
>0.35 kU_A_/L	31.4	1.9 (−0.8 to 4.5)	.002	−2.9 (−6.0 to 0.2)	.03
>0.70 kU_A_/L	26.7	0.1 (−1.9 to 2.2)	.21	−3.3 (−5.9 to −0.6)	.21

	GM, ECRHS I	GM ratio (95% CI), ECRHS II vs I	*P* value for heterogeneity between centers	GM ratio (95% CI), ECRHS III vs I	*P* value for heterogeneity between centers
Total IgE (kU/L)	27.8	0.82 (0.75 to 0.89)	<.001	0.62 (0.56 to 0.68)	<.001

**Table E5:** Net change in IgE sensitization (>0.70 kU_A_/L) to house dust mite, grass, and cat over 20 years by birth cohort

	1964-1973 (n = 736)			1954-1963 (n = 1314)			1944-1953 (n = 1156)		
Prevalence (%)	Net change (95% CI)		Prevalence (%)	Net change (95% CI)		Prevalence (%)	Net change (95% CI)	
ECRHS I	ECRHS II vs I	ECRHS III vs I	ECRHS I	ECRHS II vs I	ECRHS III vs I	ECRHS I	ECRHS II vs I	ECRHS III vs I
House dust mite	15.0	0.3 (−1.9 to 2.4)	−1.5 (-4.2 to 1.2)	14.1	−0.9 (−2.6 to 0.8)	−4.4 (−6.4 to −2.4)	9.9	−1.3 (−2.7 to 0.0)	−2.7 (−4.5 to −0.9)
Grass	18.2	1.7 (−0.8 to 4.2)	−0.7 (−3.7 to 2.4)	13.8	0.1 (−1.6 to 1.7)	−2.2 (−4.3 to −0.2)	11.4	−1.6 (−3.2 to 0.0)	−3.5 (−5.3 to −1.7)
Cat	7.7	1.0 (−1.2 to 3.1)	−0.1 (−2.3 to 2.1)	5.8	−0.3 (−1.5 to 0.9)	−0.8 (−2.2 to 0.7)	5.9	−0.3 (−1.6 to 1.0)	−2.3 (−3.6 to −1.0)
House dust mite, grass, or cat	29.5	1.2 (−1.7 to 4.1)	−2.3 (−6.0 to 1.4)	24.1	−0.6 (−2.7 to 1.6)	−5.4 (−7.9 to −2.9)	19.6	−2.2 (−4.2 to −0.3)	−5.4 (−7.8 to −3.1)

**Table I:** Net change in IgE sensitization to house dust mite, grass, and cat and total IgE levels over 20 years (n = 3206)

	Prevalence (%), ECRHS I	Net change (95% CI), ECRHS II vs I	*P* value for heterogeneity between centers	Net change (95% CI), ECRHS III vs I	*P* value for heterogeneity between centers
House dust mite					
>0.35 kU_A_/L	16.6	−0.7 (−2.2 to 0.9)	.051	−4.3 (−6.0 to −2.6)	.71
>0.70 kU_A_/L	13.1	−0.7 (−1.9 to 0.4)	.63	−3.1 (−4.5 to −1.7)	.21
Grass					
>0.35 kU_A_/L	17.0	0.5 (−1.0 to 2.0)	.048	−0.6 (−2.5 to 1.3)	.009
>0.70 kU_A_/L	14.2	0.0 (−1.3 to 1.3)	.48	−2.2 (−3.8 to −0.6)	.97
Cat					
>0.35 kU_A_/L	8.8	−0.9 (−2.1 to 0.3)	.14	−2.1 (−3.6 to −0.7)	.09
>0.70 kU_A_/L	6.4	0.0 (−1.0 to 1.1)	.15	−1.1 (−2.2 to 0.1)	.04
House dust mite, grass, or cat					
>0.35 kU_A_/L	29.4	0.1 (−2.0 to 2.1)	.003	−4.6 (−7.0 to −2.1)	.03
>0.70 kU_A_/L	24.2	−0.6 (−2.2 to 1.0)	.11	−4.6 (−6.6 to −2.6)	.17

	GM, ECRHS I	GM ratio (95% CI), ECRHS II vs I	*P* value for heterogeneity between centers	GM ratio (95% CI), ECRHS III vs I	*P* value for heterogeneity between centers
Total IgE (kU/L)	29.8	0.84 (0.78 to 0.90)	<.001	0.63 (0.58 to 0.68)	<.001

**Table II:** Net change in IgE sensitization (>0.35 kU_A_/L) to house dust mite, grass, and cat and total IgE levels (kilounits per liter) over 20 years by year-of-birth cohort

	1964-1973 (n = 736)			1954-1963 (n = 1314)			1944-1953 (n = 1156)		
Prevalence (%) or GM	Net change (95% CI)		Prevalence (%) or GM	Net change (95% CI)		Prevalence (%) or GM	Net change (95% CI)	
ECRHS I	ECRHS II vs I	ECRHS III vs I	ECRHS I	ECRHS II vs I	ECRHS III vs I	ECRHS I	ECRHS II vs I	ECRHS III vs I
House dust mite	18.6	−0.6 (−3.0 to 1.8)	−4.1 (−6.7 to −1.5)	17.2	0.2 (−1.9 to 2.4)	−4.5 (−6.9 to −2.1)	13.8	−2.0 (−3.9 to −0.1)	−4.3 (−6.6 to −1.9)
Grass	20.6	3.3 (0.4 to 6.2)	1.5 (−1.8 to 4.9)	15.9	0.5 (−1.4 to 2.3)	−0.1 (−2.5 to 2.3)	15.4	−1.9 (−3.8 to 0.0)	−3.2 (−5.3 to −1.0)
Cat	10.5	0.2 (−2.2 to 2.6)	−0.7 (−3.5 to 2.0)	8.3	−1.4 (−2.9 to 0.1)	−2.0 (−3.6 to −0.3)	8.1	−1.2 (−2.7 to 0.2)	−3.6 (−5.2 to −2.0)
House dust mite, grass, or cat	33.5	1.9 (−1.3 to 5.1)	−2.1 (−6.1 to 1.9)	28.7	1.1 (−1.6 to 3.7)	−4.1 (−7.2 to −1.1)	26.5	−3.0 (−5.6 to −0.3)	−7.4 (−10.4 to −4.3)
Total IgE	29.9	0.81 (0.72 to 0.91)	0.61 (0.54 to 0.68)	31.3	0.85 (0.78 to 0.92)	0.61 (0.56 to 0.67)	27.9	0.84 (0.78 to 0.92)	0.68 (0.61 to 0.75)

## Methods

Statistical analyses were performed with Stata software (version 13, StataCorp LP).

### Laboratory bias (duplicate measurements)

To assess the effects of potential laboratory bias on the prevalence of IgE sensitization and the mean of total IgE estimates, we conducted duplicate assays on 794 samples (tested at ECRHS I, stored, and tested at ECRHS II) and 475 samples (tested at ECRHS II, stored, and tested at ECRHS III). CIs for Cohen κ statistics for each comparison between 2 measurements of the same sample were computed by using the kap command in Stata together with delta method SEs by using the normalizing and variance-stabilizing transformation ln(1-κ) (see Table E1).

### Elimination of laboratory bias

To correct our estimates for laboratory bias, we included the following in the models:

• the 3 main-assessment assays for each participant (GMs or odds for each combination of center and ECRHS);
• 4 extra parameters (GM ratios or odds ratios) regarding the paired method-comparison assays: 2 indicating an assay's membership in the 2 method-comparison studies and 2 indicating that an assay was carried out by using the ECRHS II or III methods, respectively, instead of the ECRHS I methods.

#### Analysis of outcomes

To determine the difference in prevalence of sensitization and GM ratios of total IgE levels between surveys, we used the ‘margins’ and ‘nlcom’ commands and the ‘regpar’ add-on package,^E1^ as required.

### Inverse sampling probability weighted estimation

Inverse sampling probability weights were used to standardize the estimation from the population with data on IgE assays in all 3 ECRHSs to a target population of participants with data on IgE assays from ECRHS I, which was randomly sampled from the general adult population in different European and Australian centers.

The inverse sampling probability weights were calculated by using a logistic regression model,^E2^ with a separate set of parameters for each center with any IgE data responders, predicting response to all 3 surveys from baseline characteristics adapted from the response regression model of Jarvis et al.^E3^ The parameters for each center were baseline odds; an exponential per-decade odds ratio for age at January 1, 1992; an odds ratio for female sex (compared with a baseline of male sex); odds ratios for self-reported smoking status at ECRHS I (exsmoker and current smoker compared with a baseline of never smoker); an odds ratio for wheeze at ECRHS I; an odds ratio for waking with shortness of breath at ECRHS I; and an odds ratio for IgE sensitization to house dust mite, cat, or grass at ECRHS I. When we meta-analyzed the parameters using random-variable-effects meta-analysis,^E4^ we found that participants who have taken part in all 3 phases of the study were slightly older, less likely to be smokers, and less likely to have reported shortness of breath than participants who did not have serum IgE in all 3 surveys (Table E2).

Use of inverse sampling probability weights to standardize the estimates to the target population in ECRHS I seemed to work, as indicated by a Somers' D of the response-propensity score^E5^ with respect to a response of 0.008 when inverse sampling probability weighted versus one of 0.239 when unweighted.
